# Resting energy expenditure depends on energy intake during weight loss in people with obesity: a retrospective cohort study

**DOI:** 10.20945/2359-3997000000532

**Published:** 2022-12-01

**Authors:** Tomoko Handa, Takeshi Onoue, Tomoko Kobayashi, Eri Wada, Ayaka Hayase, Tamaki Kinoshita, Ayana Yamagami, Yoshinori Yasuda, Shintaro Iwama, Yohei Kawaguchi, Takashi Miyata, Mariko Sugiyama, Hiroshi Takagi, Daisuke Hagiwara, Hidetaka Suga, Ryoichi Banno, Motomitsu Goto, Hiroshi Arima

**Affiliations:** 1 Nagoya University Graduate School of Medicine Department of Endocrinology and Diabetes Nagoya Japan Department of Endocrinology and Diabetes, Nagoya University Graduate School of Medicine, Nagoya, Japan; 2 Nagoya University Research Center of Health, Physical Fitness and Sports Nagoya Japan Research Center of Health, Physical Fitness and Sports, Nagoya University, Nagoya, Japan

**Keywords:** Resting energy expenditure, weight loss, energy intake

## Abstract

**Objective:**

Resting energy expenditure (REE) decreases if there is reduced energy intake and body weight (BW). The decrease in REE could make it difficult for patients with obesity to maintain decreased BW. This study aimed to investigate the correlation among changes in REE, energy intake, and BW during the weight loss process in patients with obesity.

**Materials and methods:**

We conducted a retrospective cohort study of patients hospitalized for the treatment of obesity in Japan. Patients received fully controlled diet during hospitalization and performed exercises if able. REE was measured once a week using a hand-held indirect calorimetry. Energy intake was determined by actual dietary intake.

**Results:**

Of 44 inpatients with obesity, 17 were included in the analysis. Their BW decreased significantly after 1 week (−4.7 ± 2.0 kg, P < 0.001) and 2 weeks (−5.7 ± 2.2 kg, P < 0.001). The change in REE after 1 and 2 weeks was positively correlated with the energy intake/energy expenditure ratio (r = 0.66, P = 0.004 at 1 week, r = 0.71, P = 0.002 at 2 weeks). Using a regression equation (y = 0.5257x – 43.579), if the energy intake/energy expenditure ratio within the second week was 82.9%, the REE after 2 weeks was similar to the baseline level. There was no significant correlation between the change in REE and BW.

**Conclusions:**

Our data suggest that changes in REE depend on energy intake/energy expenditure ratio and that the decrease in REE can be minimized by matching energy intake to energy expenditure, even during the weight loss process.

## INTRODUCTION

Energy balance is regulated by energy intake and energy expenditure. Energy intake is derived from food. Energy expenditure comprises resting energy expenditure (REE, approximately 60%), physical activity (approximately 30%), and diet-induced thermogenesis (approximately 10%) ( 1 , 2 ). Long-term energy intake that is greater than energy expenditure leads to weight gain and, in turn, obesity. The prevalence of obesity has been increasing globally over the last several decades ( 3 ).

To treat obesity, energy intake should be controlled at a level below energy expenditure, which can be achieved by either decreasing energy intake or increasing energy expenditure. However, people with obesity cannot easily maintain a decrease in body weight. This phenomenon is attributed to, at least in part, a decrease in REE when energy intake and body weight are reduced, as not only observed in rodents ( 4 – 7 ) but also in humans ( 8 – 11 ). Previous studies have shown that reduced REE persists as long as body weight is decreased ( 8 , 12 – 14 ). In contrast, other reports have revealed that REE can recover even if the body weight is decreased ( 15 – 19 ). However, the conditions in which REE recovers remain unclear.

To clarify whether decreased REE can recover during the weight loss process, this study aimed to investigate the correlation between changes in REE as well as energy intake and body weight in patients hospitalized for treating obesity.

## MATERIALS AND METHODS

### Study design

This retrospective cohort study was conducted at Nagoya University Hospital, Japan. The study protocol was approved by the ethics committee of Nagoya University Graduate School of Medicine (no. 2015-0225). The research was performed in accordance with the ethical principles of the Declaration of Helsinki. Informed consent was obtained in the form of an opt-out in the website.

### Patients

We examined the electronic medical record data of 44 patients hospitalized for the treatment of obesity between April 1, 2011, and May 31, 2020, at Nagoya University Hospital. The participants were aged ≥ 20 years and were not on weight loss diet, and their body mass index (BMI) was ≥ 30 kg/m^2^.

The weight loss program was conducted during hospitalization. All patients were provided with an individual diet, which was set by the attending physician upon admission. Then, energy intake was adjusted as necessary during hospitalization. Additionally, patients who could exercise received exercise therapy, which was facilitated by physical therapists.

### Data collection

The following measurements were conducted during hospitalization.

#### Body weight and composition

Body weight was measured daily in the fasting state with a calibrated digital scale (to the nearest 0.1 kg; PW-650A, TANITA Corp., Tokyo, Japan) immediately after the participants had voided in the morning. Height was measured using a wall-mounted stadiometer (to the nearest 0.1 cm; Digital Height Meter AD-6400, A&D Company, Tokyo, Japan) upon admission. Waist circumference was measured at the umbilical level upon admission.

#### REE

REE was measured using a hand-held indirect calorimetry (MedGem, HealtheTech, Inc., the USA) ( 20 – 24 ). All tests were conducted between 8:00 AM and 9:00 AM, after a 12-hour fast. Testing was performed in a quiet, softly lit, well-ventilated room, with the temperature maintained between 22 ˚C and 24 ˚C. Patients were instructed to rest in a seated position for 10 min before measurement. Patients sat upright and wore a nose clip, and a disposable mouthpiece was placed in the mouth. Then, they were asked to remain still and breathe via the mouthpiece during the test (approximately 10 min). The first 2 min were eliminated as patient acclimatization to the instrument, and a steady-state VO_2_ measurement was obtained during the next 3-8 min using a rolling boxcar methodology on reiterative sets of VO_2_ in 30 breaths ( 25 , 26 ). In total, 44, 38, and 17 patients underwent the first, second, and third measurements on days 1-3 (baseline), 5-11 (1 week), and 12-18 (2 weeks), respectively.

The predicted REE (REEp) values were obtained using the Ganpule equation, as described below ( 27 ):


$$\begin{array}{c} \text{REEp }(\text{kcal}/\text{day})=\{0.1238+(0.0481\times \text{ body weight}\\ \text{ in kg})+(0.0234\times \text{ height in cm})-(0.0138\times \text{ age })-\\ \left.{\mathrm{sex}}^{*}\right\}\times 1000/4.186 \end{array}$$

*; male = 0.5473 × 1, female = 0.5473 × 2

The equation, which was developed by the National Institute of Health and Nutrition in Japan, is more effective in predicting REE in Japanese populations compared to other formulae, including the Harris-Benedict equation ( 28 ). REEm-p was defined as the difference between the measured REE (REEm) and REEp.

#### Energy expenditure

Patients’ activities were controlled during hospitalization, and those patients who could exercise performed exercises equivalent to 60 minutes of walking per day, which were supervised by physical therapists. Patients who did not perform any exercise spent most of their time in their hospital rooms only. Considering this situation, energy expenditure was calculated as follows:

Energy expenditure (kcal/day) = REE (kcal/day) × activity factor

The activity factors were 1.3 in patients without exercise therapy and 1.5 in patients with exercise therapy ( 29 , 30 ).

#### Energy intake

The attending physician determined the indicated energy intake based on REE and physical activity, as well as the Japan Society for the Study of Obesity guidelines ( 31 ). According to the guidelines, the energy intake of patients with obesity should be 25 kcal/kg × standard body weight/day or less for 25 kg/m^2^ ≤ BMI < 35 kg/m^2^, and 20-25 kcal/kg × standard body weight/day or less for a BMI of ≥ 35 kg/m^2^. The standard body weight was calculated as height (m) × height (m) × 22. If the patient left food uneaten, this was also taken into account when calculating the actual energy intake. The energy intake/energy expenditure ratio within the first week was calculated as the average energy intake/energy expenditure ratios on days 1-7 based on the baseline REE. That within the second week was calculated as the average energy intake/energy expenditure ratios on days 8-14 based on the REE at 1 week. During hospitalization, meals were prepared by the Nagoya University Hospital kitchen. The meal composition included 55%-59% of energy as carbohydrate, 25%-28% as fat, and 1.3-1.5 g per 30 kcal as protein.

We collected data about clinical parameters such as blood pressure, blood biochemistry results, medical history, and therapy during hospitalization from the electronic medical records. Obesity-related disorders were diagnosed on the basis of the Japan Society for the Study of Obesity guidelines ( 31 ). Thyroid disease was defined as a medical history of hypothyroidism or hyperthyroidism.

### Statistical analysis

Continuous variables were expressed as mean ± standard deviation (SD) and *categorical variables* as numbers (percentages). One-way repeated-measures ANOVA followed by *post hoc* Tukey’s test was performed to determine significant differences before and after the comparison of body weight, BMI, energy intake, and REE during the weight loss program. The paired *t* -test was used to determine significant differences before and after the comparison of energy expenditure during the weight loss program. A correlation analysis was performed using Pearson correlation coefficients. JMP Pro version 15.0.0 (SAS Institute Inc., Cary, NC, the USA) was used for all statistical analyses. A *P* valueof < 0.05 was considered statistically significant.

## RESULTS

**Figure 1:**
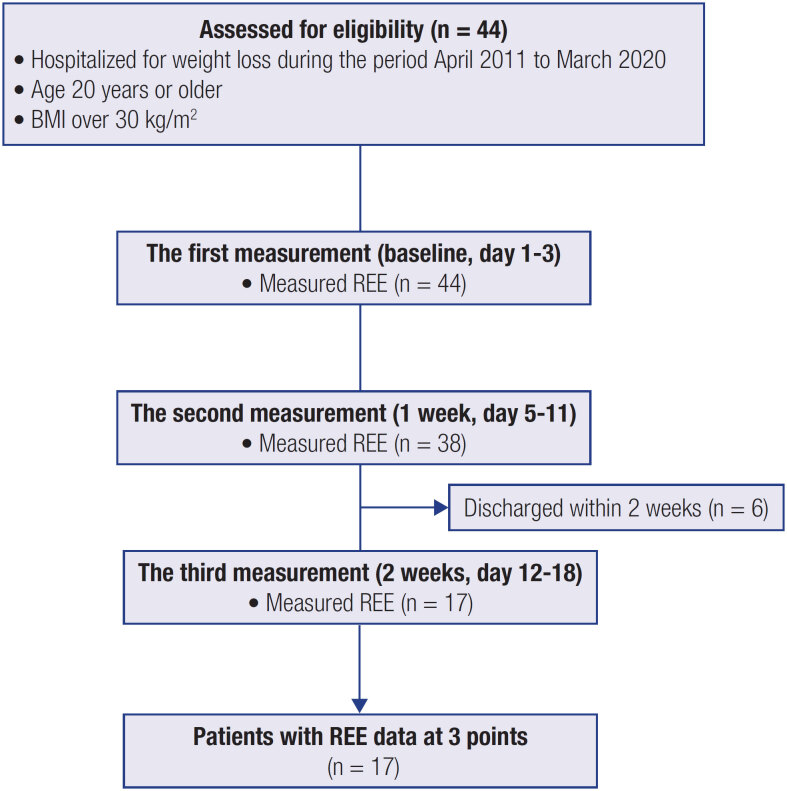
Study flowchart.

Figure 1 presents the study flowchart. In total, 44, 38, and 17 patients underwent the first (baseline), second (1 week), and third (2 weeks) REE measurements, respectively.

Table 1 shows the baseline characteristics of patients who had REE data at the three time points. In total, 12 patients were men and 5 women, with an average age of 47.6 ± 12.4 years and BMI of 42.4 ± 9.3 kg/m^2^. Approximately 70.6% of patients presented with type 2 diabetes, 94.1% with hypertension, and 88.2% with dyslipidemia.

**Table 1:** Baseline characteristics of participants

	Patients with REE data of 3 points (n = 17)
Age (years)	47.6 ± 12.4
Sex, male	12 (70.6%)
Body weight (kg)	115.0 ± 22.9
BMI (kg/m^2^)	42.4 ± 9.3
Waist circumference (cm) a	135.3 ± 9.8
Type 2 diabetes	12 (70.6%)
Hypertension	16 (94.1%)
Dyslipidemia	15 (88.2%)
Hyperuricemia	8 (47.1%)
Proteinuria	7 (41.2%)
Cardiovascular disease	3 (17.6%)
Cerebrovascular disease	1 (5.9%)
NAFLD	11 (64.7%)
OSAS, OHS	5 (29.4%)
Musculoskeletal diseases b	4 (23.5%)
Thyroid disease	0 (0%)
Oral steroids	0 (0%)
Anti-obesity drug c	1 (5.9%)
Diuretic	9 (52.9%)
SGLT-2 inhibitor	2 (11.8%)

Data were expressed as mean ± SD, or n (%) values. REE: resting energy expenditure; BMI: body mass index; NAFLD: nonalcoholic fatty liver disease; OSAS: obstructive sleep apnea syndrome; OHS: obesity hypoventilation syndrome; SD: standard deviation; SGLT-2 inhibitor: sodium-glucose co-transporter-2 inhibitor.

a n = 6. Eleven patients had missing data about the waist circumference.

b Two patents presented with gonarthrosis and another two with hip osteoarthritis.

c One patient was treated with Bofutsushosan.

Table 2 shows data about body weight, BMI, energy intake, REEm, REEp, REEm−p, and energy expenditure during the weight loss program. The body weight decreased significantly at 1 week (−4.7 ± 2.0 kg, *P* < 0.001) and 2 weeks (−5.7 ± 2.2 kg, *P* < 0.001), and 17 patients lost weight (1.8-8.7 kg at 1 week and 2.7–9.7 kg at 2 weeks from the baseline) during the program. The REEp decreased significantly at 1 week (−53.6 ± 23.4 kcal/day, *P* < 0.001) and 2 weeks (−65.6 ± 25.1 kcal/day, *P* < 0.001). In contrast, there was no significant change in REEm or REEm-p during the weight loss process.

**Table 2:** Body weight, BMI, energy intake, REEm, REEp, REEm-p, and energy expenditure during the weight loss program

	(Baseline)	(1 week)	(2 weeks)
	the first measurement	the second measurement	the third measurement
Body weight (kg)	115.0 ± 22.9	110.3 ± 22.0 **	109.3 ± 21.5 **
BMI (kg/m^2^)	42.4 ± 9.3	40.7 ± 9.0 **	40.3 ± 8.6 **
Energy intake (kcal/day)	1623 ± 292	1566 ± 303	1558 ± 192
REEm (kcal/day)	1589 ± 454	1551 ± 378	1492 ± 343
REEp (kcal/day)	1947 ± 353	1893 ± 344 **	1881 ± 339 **
REEm-p (kcal/day)	−357 ± 314	−342 ± 271	−390 ± 221
EE (kcal/day)	2257 ± 740	2203 ± 647	

BMI: body mass index; REEm: measured resting energy expenditure; REEp: predicted resting energy expenditure; REEm-p: the difference between the measured and predicted REEs at each time point; EE: energy expenditure.

** *P* < 0.01 vs baseline value.

**Figure 2:**
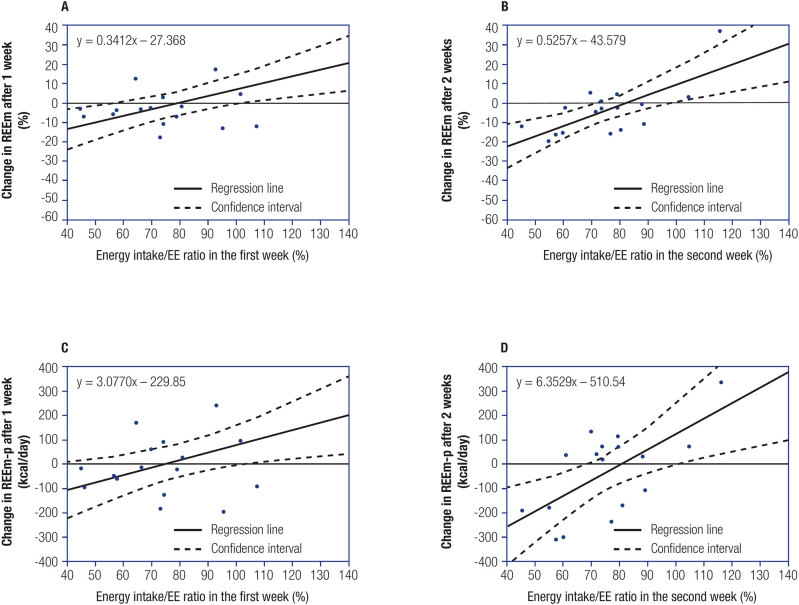
Correlation between the energy intake/energy expenditure ratio and the change in REEm or REEm-p. Correlation between the energy intake/energy expenditure ratio within the first week and the change in REEm (%) after 1 week ( **A** ) and between the energy intake/EE ratio within the second week and change in REEm (%) after 2 weeks ( **B** ). Correlation between the energy intake/EE ratio within the first week and change in REEm-p (kcal/day) after 1 week ( **C** ) and between the energy intake/EE ratio within the second week and change in REEm-p (kcal/day) after 2 weeks ( **D** ).

Figure 2A shows the correlation between the energy intake/energy expenditure ratio within the first week and the change in REEm after 1 week. Figure 2B depicts the correlation between the energy intake/energy expenditure ratio within the second week and the change in REEm after 2 weeks. The change in REEm was positively correlated with the energy intake/energy expenditure ratio (r = 0.66, *P* = 0.004 at 1 week, r = 0.71, *P* = 0.002 at 2 weeks) ( Table 3 ). The regression equation was y = 0.5257x – 43.579 at 2 weeks ( Figure 2B ). This indicates that if the energy intake/energy expenditure ratio within the second week was 82.9%, the change in REEm during the weight loss program was 0% (i.e., similar to that at baseline).

**Table 3:** Correlation analysis between the energy intake/energy expenditure ratio and the change in REEm or REEm-p

	Average	SD	r	*P* -value
Energy intake/EE ratio in the first week (%)	79.7	29.7	0.66	**0.004**
Change in REEm after 1 week (%)	−0.2	15.3	
Energy intake/EE ratio in the second week (%)	75.3	17.7	0.71	**0.002**
Change in REEm after 2 weeks (%)	−4.0	13.2		
Energy intake/EE ratio in the first week (%)	79.7	29.7	0.58	**0.015**
Change in REEm-p after 1 week (kcal/day)	15.3	157.8		
Energy intake/EE ratio in the second week (%)	75.3	17.7	0.64	**0.005**
Change in REEm-p after 2 weeks (kcal/day)	−32.1	175.5		

REEm: measured resting energy expenditure; REEp: predicted resting energy expenditure; REEm-p: difference between the measured and predicted REEs at each time point; EE: energy expenditure; r: correlation coefficient. *P* -values of < 0.05 are presented in bold text.

Figure 2C shows the correlation between the energy intake/energy expenditure ratio within the first week and change in REEm-p after 1 week. The change in REEm-p was positively correlated with the energy intake/energy expenditure ratio (r = 0.58, *P* = 0.015) ( Table 3 ). Figure 2D shows the association between the energy intake/energy expenditure ratio within the second week and change in REEm-p at 2 weeks after the start of the program. The change in REEm-p was positively correlated with the energy intake/energy expenditure ratio (r = 0.64, *P* = 0.005) ( Table 3 ).

**Supplementary Figure S1:**
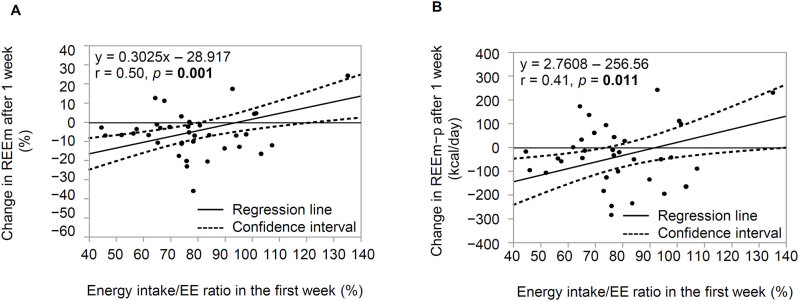
Correlation between the energy intake/energy expenditure ratio and the change in REEm or REEm-p in 38 patients. Correlation between the energy intake/energy expenditure ratio within the first week and change in REEm (%) after 1 week ( **A** ) and change in REEm-p (kcal/day) after 1 week ( **B** ) in 38 patients, in whom REE was assessed at the first and second measurements (not at the third measurement). REEm, measured resting energy expenditure; REEp, predicted resting energy expenditure; REEm-p, difference between the measured and predicted REEs at each time point; EE, energy expenditure; r, correlation coefficient. *P* -values of < 0.05 are presented in bold text.

**Supplementary Figure S2:**
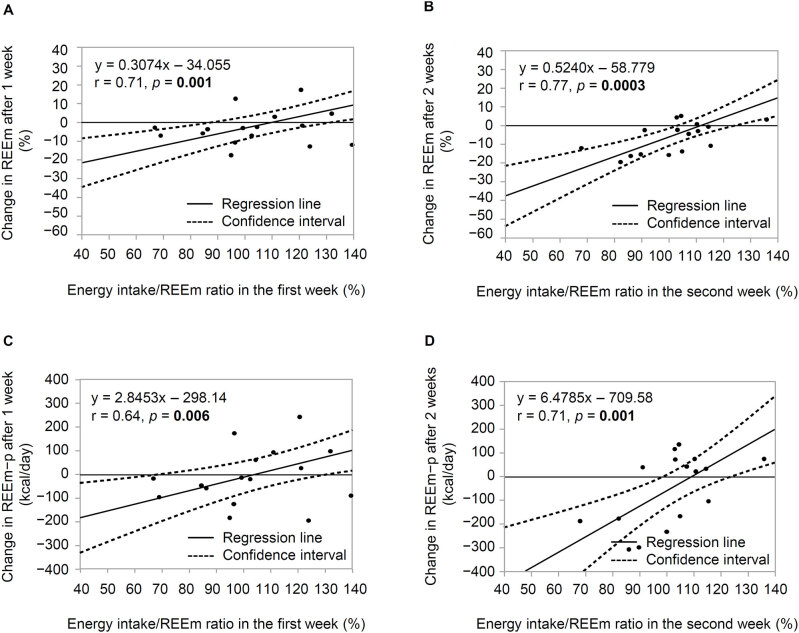
Correlation between the energy intake/REEm ratio and the change in REEm or REEm-p. Correlation between the energy intake/REEm ratio within the first week and change in REEm (%) after 1 week ( **A** ) and between the energy intake/REEm ratio within the second week and change in REEm (%) after 2 weeks ( **B** ). Correlation between the energy intake/REEm ratio within the first week and change in REEm-p (kcal/day) after 1 week ( **C** ) and between the energy intake/REEm ratio within the second week and change in REEm-p (kcal/day) after 2 weeks ( **D** ).

Moreover, significant correlation was also observed in 38 patients, in whom REE was assessed at the first and second measurements (Supplementary Figures 1A and B ). We also examined the correlation between the change in REE and ratio of energy intake to measured REE, instead of the estimated energy expenditure, and found a significant correlation (Supplementary Figure 2 ).

There was no significant correlation between the change in body weight and that in REEm, REEm-p, and energy intake/energy expenditure ratio ( Table 4 ).

**Table 4:** Correlation analysis between the change in body weight and that in REEm, REEm-p, and energy intake/energy expenditure ratio

	Average	SD	r	*P* -value
Change in body weight after 1 week (%)	−4.1	1.5	0.12	0.646
Change in REEm after 1 week (%)	−0.2	15.3		
Change in body weight after 2 weeks (%)	−4.9	1.3	−0.13	0.621
Change in REEm after 2 weeks (%)	−4.0	13.2		
Change in body weight after 1 week (%)	−4.1	1.5	0.06	0.831
Change in REEm−p after 1 week (kcal/day)	15.3	157.8		
Change in body weight after 2 weeks (%)	−4.9	1.3	−0.27	0.287
Change in REEm−p after 2 weeks (kcal/day)	−32.1	175.5		
Change in body weight after 1 week (%)	−4.1	1.5	−0.03	0.911
Energy intake/EE ratio in the first week (%)	79.7	29.7		
Change in body weight after 2 weeks (%)	−4.9	1.3	0.005	0.986
Energy intake/EE ratio in the second week (%)	75.3	17.7		

REEm: measured resting energy expenditure; REEp: predicted resting energy expenditure; REEm-p: difference between the measured and predicted REEs at each time point; EE: energy expenditure; r: correlation coefficient. *P* -values of < 0.05 are presented in bold text.

## DISCUSSION

This retrospective cohort study investigated changes in REE, energy intake, and body weight in patients hospitalized for the treatment of obesity. Our data showed that the changes in REE were significantly correlated with the energy intake/energy expenditure ratio, but not with body weight.

During weight loss, the REE decreases at an extent greater than that expected from changes in body weight. This phenomenon is referred to as metabolic adaptation or adaptive thermogenesis ( 8 , 9 ). Our data showed that the change in the difference between the measured and predicted REEs after starting the weight loss program was significantly correlated with the energy intake/energy expenditure ratio, as reported in a previous report ( 32 ). This indicates that severe caloric restriction can result in greater metabolic adaptation.

Our data showed that the change in REE was correlated with the energy intake/energy expenditure ratio during not only the first week but also the second week of the weight loss program. Maintaining decreased body weight is challenging among patients with obesity who present with a reduced REE ( 33 – 35 ). Therefore, if possible, REE should be recovered without increasing body weight. Previous studies revealed that intermittent caloric restriction prevented the reduction in REE associated with caloric restriction while achieving long-term weight loss ( 18 , 19 ). However, loosening caloric restriction may result in weight gain. Our data showed that if the energy intake/energy expenditure ratio was set at more than 82.9% at 1 week after starting weight loss, when REE decreased, the REE at 2 weeks could recover to baseline levels. Therefore, patients with obesity could recover their REE without increasing body weight during the weight loss program.

Several studies have revealed that the changes in REE were positively correlated with changes in body weight ( 17 , 36 ). However, we found no significant correlation between them. In this study, 9 (52.9%) of 17 patients were treated with diuretics. In addition, one patient with renal failure and two with heart failure were included in this study. The body weight of these patients could decrease with salt restriction during hospitalization; thus, weight change might not reflect energy balance exclusively.

The current study had several limitations. First, since this was a retrospective study, the number of patients with REE data at all time points was limited. Second, the accuracy of energy expenditure estimation is limited because the estimation of physical activity is based on the degree of activity during hospitalization, not on measurement by activity meters or other devices. Third, as all participants were hospitalized, the analysis commonly lasted for only 2 weeks.

Thus, a prospective study with a larger number of patients and with a longer duration must be performed to examine if setting energy intake based on REE was effective in maintaining decreased body weight in patients with obesity.

In conclusion, our data suggest that changes in REE depend on energy intake/energy expenditure ratio and that the decrease in REE can be minimized by matching energy intake to energy expenditure, even during the weight loss process.
